# First case report of pulmonary nocardiosis caused by *Nocardia mexicana*

**DOI:** 10.1099/jmmcr.0.005054

**Published:** 2016-08-30

**Authors:** Tomokazu kuchibiro, Takeshi Ikeda, Hirotaka Nakanishi, Yukiko Morishita, Katsuyuki Houdai, Junko Ito, Tohru Gonoi

**Affiliations:** ^1^​ Department of Clinical Laboratory, Naga Municipal Hospital, 1282 Uchita, Kinokawa, Wakayama, 649-6414, Japan; ^2^​ Department of Respiratory Medicine, Naga Municipal Hospital, 1282 Uchita, Kinokawa, Wakayama, 649-6414, Japan; ^3^​ Medical Mycology Research Center, Chiba University, 1-8-1 Inohana, Chuoku, Chiba City, Chiba, 260-8673, Japan

**Keywords:** pulmonary nocardiosis, *Nocardia mexicana*, 16S rRNA

## Abstract

**Introduction:**

*Nocardia* species usually cause opportunistic infections, and the frequency of these infections is increasing owing to the growing population of immunocompromised hosts. However, *Nocardia* species may sometimes cause an infection disease in immunocompetent hosts. *Nocardia mexicana* infections are the least common and are very rare.

**Case presentation:**

Herein, we report the first case of a pulmonary infection with *N. mexicana* in a 61-year-old Japanese woman with a history of hyperlipidaemia and bronchiectasis and a 6-month history of non-productive hacking cough. A sample of bronchial lavage fluid obtained by bronchofiberscopy showed filamentous branching gram-positive rods and acid-fast filamentous branching rods, and a colony of suspected *Nocardia* was cultured. Based on 16S rRNA, *gyrB, rpoB*, *secA1* and *hsp65 *gene sequence analyses and biochemical and physiological properties, the strain was identified as *N. mexicana.* The strain was resistant to the antimicrobial agents amoxicillin-clavulanic acid, clarithromycin, minocycline, gentamycin, tobramycin, ciprofloxacin and trimethoprim-sulfamethoxazole. The patient was treated with biapenem followed by intravenous amikacin and oral linezolid.

**Conclusion:**

Despite its rarity, the species require attention owing to the existence of multidrug-resistant strains.

## Introduction

*Nocardia* species have a worldwide distribution and are commonly found as saprophytes in soil or water. As aerobic actinomycetes, *Nocardia* species are gram-positive, filamentous, beaded bacteria; they are weakly acid-fast and slow growing (Lerner, 1996). *Nocardia* species usually cause opportunistic infections, whose prevalence has been increasing owing to the growing population of immunocompromised hosts. The most common predisposing factors for opportunistic nocardiosis are long-term steroid use, neoplastic disease and human immunodeficiency virus infection (HIV) (Brown-Elliott *et al*., 2006). Because the pathogenicity of *Nocardia* species is low, there are fewer cases of infections in immunocompetent patients. However, the infection case in the immunocompetent patients by the *Nocardia *species is often reported. Only 15 *Nocardia* species were classified in 1995; today, >100 species have been classified owing to the development of molecular technologies (http://www.ncbi.nlm.nih.gov/Taxonomy/Browser/wwwtax.cgi). Amongst the *Nocardia *species,* Nocardia mexicana* is not frequently isolated, and infections caused by this species are rarely reported. We report the first case of pulmonary infection caused by *N. mexicana* in an immunocompetent patient.

## Case report

A 61-year-old Japanese woman was referred from another institution with a 6-month history of non-productive hacking cough, which required further investigation. The patient had hyperlipidaemia and bronchiectasis. However, it was not the underlying disease that caused immunodeficiency, not receiving any steroid therapy and immunosuppressive agents, and the serum examination of HIV was negative, making her an immunocompetent host. She was not an alcoholic but had a smoking history of 20 packs per year. Her work exposed her to organic solvents for many years, and her social history was unremarkable. There was no recent history of foreign travel. Shadows observed to right lower lobe and left lower lobe on the chest X-ray film indicated a probable diagnosis of pneumonia; therefore, garenoxacin therapy was initiated. However, the cough worsened. Culture examination had not been carried out in the other institution.

## Investigations

**Fig. 1.**
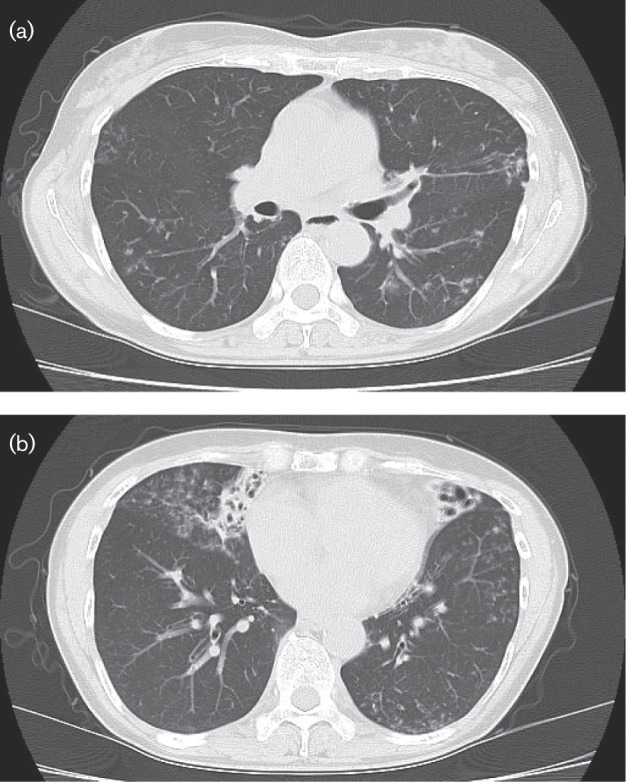
(a) Chest CT on admission showed scattered nodules in the inferior lobe lung. (b) Chest CT showed bronchiectasis in the middle lobe lung.

The patient was referred to Naga Municipal Hospital for further evaluation and treatment. The haematological tests performed on admission revealed a C-reactive protein level of 13.3 mg l^−1^ and a white blood cell count of 15.19×10^6^ l^−1^, with 71 % neutrophils. Because the chest computed tomography (CT) scans showed scattered nodules in the inferior lobe of the lung and bronchiectasis in the middle lobes (Fig. 1), pulmonary disease was clinically diagnosed. We did not perform cerebrospinal fluid examination or brain CT/magnetic resonance imaging (MRI).

**Fig. 2.**
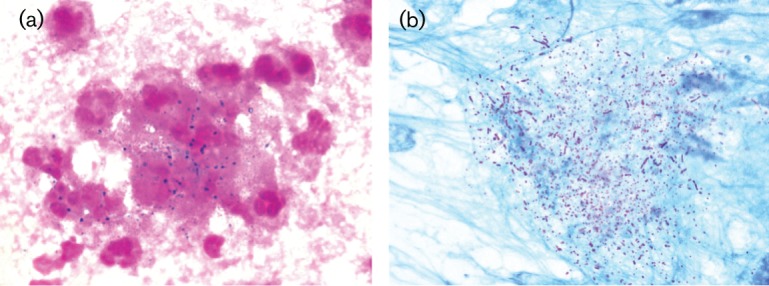
(a) Gram stain (×1000) of bronchial lavage fluid showing gram-positive, branching, beaded filaments. (b) Modified acid-fast Kinyoun stain (×1000) of bronchial lavage fluid showing filamentous red-stained partially acid-fast rods.

Obtaining sputum samples was not possible; therefore, the bronchial lavage fluid was collected by bronchofiberscopy. Gram staining of the sample showed filamentous, branching gram-positive rods (Fig. 2a). Modified Kinyoun acid-fast staining using a weak acid (1 % sulphuric acid–water) showed partially acid-fast filamentous, branching rods that were identified to be belonging to the *Nocardia* genus based on their morphologies and staining properties (Fig. 2b). After 48 h of incubation at 35 °C in an aerobic atmosphere, small, rough, dry and non-haemolytic chalky white colonies emitting an earthy odour were observed on sheep blood agar.

## Diagnosis

For species identification, 16S rRNA gene sequencing using universal eubacterial primers was performed. A blast search (www.ncbi.nlm.nih.gov/BLAST) for the sequence obtained was performed using the taxonomy browser of the National Center of Biotechnology Information (NCBI). The first 500 bp of the 16S rRNA sequence was searched against the NCBI GenBank database; the search revealed a 99.6 % (480/482 bp) match to that of a *N. mexicana* sequence published by Rodríguez-Nava *et al.* (Rodríguez-Nava *et al*., 2004). The 1474-bp fragment of the 16S rRNA sequence (GenBank accession number LC060739) showed 98.4 % homology (1451/1474 bp) with that of the type strain of *N. mexicana*.

**Fig. 3.**
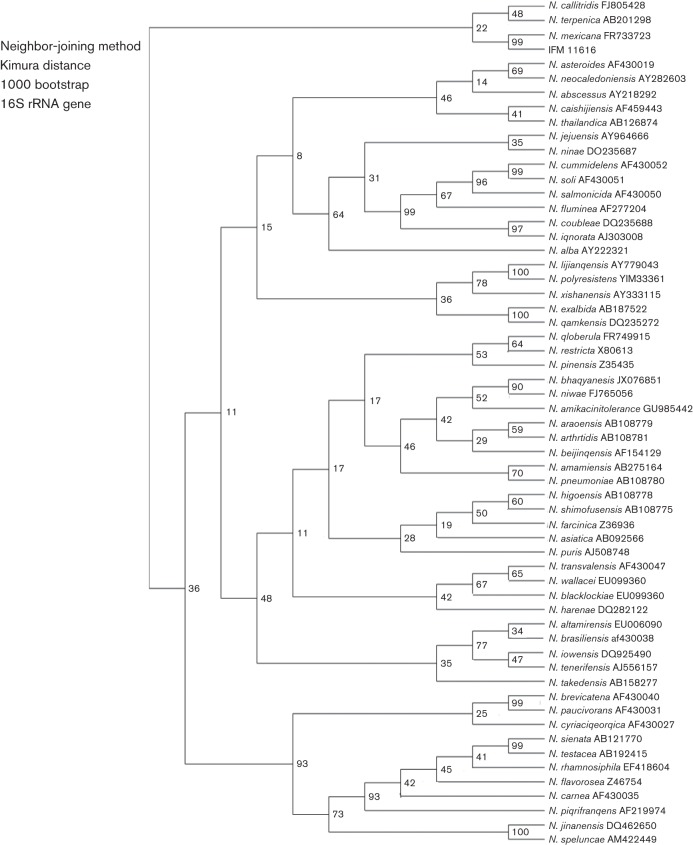
Phylogenetic trees based on the 16S rRNA, *gyrB* and *rpoB* sequences from IFM 11616 and the most closely related *Nocardia*. These trees were based on a comparison of stretches of 1474 nucleotides of the 16S rRNA gene, 855 nucleotides of the *gyrB* gene and 302 nucleotides of the *rpoB* gene. Evolutionary trees were inferred by the neighbour-joining method with bootstrap resampling (1000 replicates).

To supplement the results of the 16S rRNA gene sequencing, additional *rpoB*, *secA1, gyrB* and *hsp65* sequences of this strain (IFM 11616) were obtained and compared with those of the *N. mexicana* from the DNA Data Bank of Japan (DDBJ) (http://www.ddbj.nig.ac.jp/searches-e.html). Partial sequence analysis of these housekeeping genes revealed 99.1 % homology for *hsp65* (426/430 bp), 99.1 % for *secA1* (426/430 bp) and 94.0 % for *rpoB* (286/302 bp) between the isolated strain and *N. mexicana*. The results of the sequence analysis suggested most closeness with *N. mexicana*, based on the information obtained from the DDBJ database. The *gyrB* gene showed 93.3 % (798/855 bp) homology with that of *N. mexicana*, but the most closely related bacterial gene was that of *Nocardia transvalensis,* which showed 96.3 % sequence homology (825/857 bp). According to the phylogenetic trees constructed based on the 16S rRNA, *gyrB* and *rpoB* sequences using the neighbour-joining method (Fig. 3) and the unweighted pair group method with arithmetic mean (data not shown), IFM 11616 was most closely related to the *N. mexicana* genotype among the *Nocardia* species. In addition, the characteristics of IFM 11616 were similar to those of *N. mexicana, *whose peculiar physicochemical characteristics differ from those of other *Nocardia* species (Rodríguez-Nava *et al*., 2004) (Table 1). Based on this comprehensive analysis, we inferred that IFM 11616 belongs to *N. mexicana.*

**Table 1.** Physiological chemical characteristics of *N. mexicana* CIP 108295T, clinical isolate (IFM 11616) and reference *Nocardia* strains

Test	Characteristics of strains						
	1	2	3	4	5	6	7
Growth on carbon sourced
l-Arabinose	+	+	+	−	−	−	−
d-Galactose	+	+	+	−	−	+	+
d-Glucose	+	+	+	+	+	+	+
Maltose	−	−	−	−	−	−	−
d-Mannitol	+	+	+	nd	nd	nd	nd
Mannose	+	−	+	−	−	−	−
l-Rhamnose	+	+	−	−	−	−	−
Sorbitol	+	+	−	−	−	−	−
Growth on Bennett agar at:
45 °C	−	−	−	−	+	−	−
Urease	+	+	+	+	+	+	+
Decomposition of
Adenine	+	+	−	−	−	−	+
Casein	−	−	−	−	−	+	+
Hypoxanthine	+	+	+	−	−	+	+
Tyrosine	w	−	−	−	−	+	+
Xanthine	−	+	+	−	−	−	−

Strains: 1, *N. mexicana* CIP 108295^T^ (1); 2,* N. mexicana* CIP 108295^T^ (our result); 3, clinical isolate (IFM 11616); 4, *Nocardia asteroides* ATCC 19247^T^; 5, *Nocardia farcinica *ATCC3318^T^; 6, *Nocardia brasiliensis *ATCC 19296^T^; 7, *Nocardia pseudobrasiliensis* DSM 44290^T^. Reactions: −, negative; +, positive; w, weak; nd, not determined.

## Treatment

The antimicrobial susceptibility of IFM 11616 was tested using a broth microdilution method according to the Clinical and Laboratory Standards Institute (CLSI) standard M24-A2 guidelines (CLSI, 2011). The minimal inhibitory concentrations were determined using a dry plate (Eiken Chemical) incubated at 37 °C for 72 h, and the results were interpreted according to breakpoints established for the *Nocardia* genus. Based on the results of this analysis, the clinical IFM 11616 isolate was found to be resistant to most of the antibiotics tested: amoxicillin-clavulanic acid (16/32 µg ml^−1^), clarithromycin (> 8 µg ml^−1^), minocycline (MINO, 4 µg ml^−1^), doxycycline (4 µg ml^−1^), gentamycin (>16 µg ml^−1^), tobramycin (>16 µg ml^−1^), ciprofloxacin (>8 µg ml^−1^) and trimethoprim-sulphamethoxazole (TMP/SMX, >8/152 µg ml^−1^). It showed *in vitro* susceptibility to cefotaxime (8 µg ml^−1^), ceftriaxone (<2 µg ml^−1^), cefepim (2 µg ml^−1^), amikacin (AMK, <4 µg ml^−1^), imipenem (<0.5 µg ml^−1^), meropenem (0.5 µg ml^−1^) and linezolid (LZD, 4 µg ml^−1^).

The patient was initially treated with oral TMP/SMX (160/800 mg, thrice daily) and MINO (200 mg/day), but her condition deteriorated and she had to be hospitalized. Based on the results of the antimicrobial susceptibility test, the therapy was switched to intravenous biapenem (BIPM) (0.9 g/day) administration for 4 weeks. The patient was then treated with alternating cycles of the following: oral LZD for 2 weeks and intravenous AMK (600 mg/48 h) for 3 weeks. The total therapy period comprising administration of these two drugs was 5 months. The LZD dose was maintained at 1200 mg/day during this period; however, considering the findings of therapeutic drug monitoring, the AMK dosage was changed to 800 mg/48 h after 1 cycle at 600 mg/48 h.

## Outcome and follow-up

We chose alternating oral LZD and intravenous AMK administration for outpatient follow-up therapy. After a follow-up, it appeared that the infectious disease had been treated. However, 3 months after the completion of the 6-month antimicrobial treatment, the patient showed a recurrence of symptoms characteristic of the infection. The bronchial lavage fluid was again collected and cultured, but only *N. mexicana* was isolated in the culture. Therefore, we diagnosed the condition as recurrence of pulmonary nocardiosis. Subsequently, antimicrobial treatment was resumed. Because the patient was determined to require further long-term antimicrobial treatment, BIPM was administered, followed by readmission for 6 weeks. The symptoms eventually improved, and we aim to continue our follow-up with AMK and LZD treatment for 12 months.

## Discussion

Clinically, nocardiosis is a rare and potentially life-threatening infection caused by a gram-positive bacterium. *Nocardia* species are opportunistic pathogens that most commonly affect immunocompromised patients, although approximately one-third of the patients are immunocompetent (Beaman & Beaman, 1994 Nocardiosis presents as a pulmonary, primary, cutaneous and disseminated disease. The lungs are the most common primary site of infection (Lederman & Crum, 2004). In this case, although we investigated the immune status, it was thought that the patient had normal level of immunocompetence for steroid independence and that she was HIV negative. Thus, this was a case of a rare infectious disease in a person who had a normal immune status without any underlying condition that would compromise this immune status. The comparison of *N. mexicana *with other *Nocardia* species is not possible in this case, but it may be necessary to evaluate the pathogenicity of *N. mexicana *as pulmonary nocardiosis in this case was caused in an immunocompetent patient. *Nocardia* species are also known to cause infections in the central nervous system, skin and other locations (Brown-Elliott *et al.*, 2006). *Nocardia* cerebral abscess generally occurs in immunocompromised patients, and critical cases of infection in immunocompetent patients are extremely rare. Because no symptoms other than respiratory ones were observed in the present case, we did not perform cerebrospinal fluid examination or brain CT/MRI. However, care should be exercised because many infectious diseases of the central nervous system have been reported in pulmonary nocardiosis (Flateau *et al*., 2013; Kim *et al*., 2015). *N. mexicana* was first described by Rodríguez-Nava *et al*. who reported three human mycetoma cases from Mexico (Rodríguez-Nava *et al*., 2004). To the best of our knowledge, this is the first reported case of pulmonary infection caused by *N. mexicana*. Bacteria from the *Nocardia* genus are saprophytes and are found in soil environments worldwide. Moreover, reports of infection in cattle in Australia (Owen *et al*., 2015) suggest that *N. mexicana* may exist all over the world, in addition to Mexico. Based on these reports and our findings, we believe that *N. mexicana* may be present in Japan. Because the patient in this case does not have a recent history of foreign travel, the possibility of her being infected in Japan is thoughtworthy. However, the source of infection in this case remained unclear because the patient did not have a reliable history of contact with soil environments and animals.

The use of molecular methods for the identification of members of the *Nocardia* genus at the species level has increased recently. Identification of aerobic actinomycetes at the genus level is considered reliable if there is a ≥99.6 % match with the first 500 bp of the 16S rRNA gene sequence, according to the CLSI guideline MM18-A (CLSI, 2008). The first 500 bp of the 16S rRNA sequence of IFM 11616 isolated from the bronchial lavage fluid of the patient satisfied this threshold. However, bacteria with a sequence homology >98.7 % are regarded as identical (Stackebrandt *et al.*, 2006); the 1474 bp of the 16S rRNA sequence of IFM 11616 showed 98.4 % homology with that of *N. mexicana*, indicating a low probability of its identification to the *Nocardia* species level. However, 16S rRNA has a low mutation rate in evolution, which occasionally distinguishes closely related species from each other. *Nocardia* species identification may be difficult due to complex and rapid changes in the 16S rRNA sequence, because of which 16S rRNA sequence analysis is insufficient to distinguish between many closely related species in this case. In such cases, the sequence analysis of other housekeeping genes (*gyrB, rpoB, hsp65* and *secA1*) is used for distinguishing between different *Nocardia* species (McTaggart *et al.*, 2010; Takeda *et al*., 2010; Carrasco* et al*., 2013). We identified IMF11616 to be closely related to *N. mexicana* based on the analysis of the sequences of 16S rRNA and four housekeeping genes; phylogenetic trees were also constructed based on the sequence analysis of three genes. The method of identification of *Nocardia* species using 16S rRNA gene sequencing analysis is the gold standard, but in cases where differentiation of bacterial species based on 16S rRNA gene sequencing analysis becomes difficult, the analysis of the sequences of other multiple housekeeping genes and the construction of phylogenetic trees prove useful. However, when we analysed the *gyrB* sequence, the best match in DDBJ was found to be the *gyrB* gene of *N. transvalensis*. The absence of the *gyrB* sequence in the type strain of *N. mexicana* submitted to DDBJ may be the reason for this result. Therefore, when a gene sequence is being analysed, it is always important to consider that it might not be included in the database being searched.

*N. mexicana* has peculiar physicochemical characteristics that differ from those of other *Nocardia* species, and the characteristics of IFM 11616 were similar to those of *N. mexicana.* The conventional physicochemical testing used for the accurate identification of some *Nocardia* species has been recommended against recently. However, this method was used for the identification of *N. mexicana* in this study because the characteristics of *N. mexicana* were very specific. The antimicrobial susceptibility tests showed that IFM 11616 was resistant to many types of antibiotics. Rodríguez-Nava *et al.* reported that *N. mexicana* is multidrug resistant (Rodríguez-Nava *et al*., 2004), which is consistent with the results of this study. Therefore, it is likely that *N. mexicana* is a multidrug-resistant species of the *Nocardia* genus. The cases of *N. mexicana* infection are very rare not only in Japan but also around the world. Therefore, it is necessary to accumulate clinical reports on such cases.

Traditionally, TMP/SMX and MINO are among the first-line antibiotics chosen for pulmonary nocardiosis treatment (Burgert, 1999; Saubolle *et al*., 2003; Minero *et al*., 2009). However, the strain isolated in the present case was resistant to TMP/SMX and MINO; therefore, the drugs did not show any clinical efficacy. We chose BIPM based on the susceptibility results, and this therapy was beneficial to the patient. Long-term oral therapy is usually necessary, but the only oral drug that the strain was susceptible to *in vitro* was LZD. In addition, increasing evidence supports its efficacy in the long-term treatment of nocardiosis (Moylett *et al*., 2003; Shen *et al*., 2011; Tanioka *et al.*, 2012); therefore, LZD may be a suitable alternative. However, anaemia and thrombocytopenia are frequently associated with prolonged LZD treatment, and rare but severe adverse events such as lactic acidosis, neuropathy and visual impairment have been reported (Rivero *et al*., 2008; Shen *et al*., 2011).

In conclusion, to our knowledge, this is the first report on a case of pulmonary nocardiosis caused by *N. mexicana*. Despite its rarity, the species require attention owing to the existence of its multidrug-resistant strains.
